# Climatic change drives dynamic source–sink relationships in marine species with high dispersal potential

**DOI:** 10.1002/ece3.7204

**Published:** 2021-02-16

**Authors:** Catarina N. S. Silva, Emma F. Young, Nicholas P. Murphy, James J. Bell, Bridget S. Green, Simon A. Morley, Guy Duhamel, Andrew C. Cockcroft, Jan M. Strugnell

**Affiliations:** ^1^ Centre for Sustainable Tropical Fisheries and Aquaculture James Cook University Townsville Qld Australia; ^2^ British Antarctic Survey Cambridge UK; ^3^ Department of Ecology La Trobe University Melbourne Vic. Australia; ^4^ School of Biological Sciences Victoria University of Wellington Wellington New Zealand; ^5^ Institute for Marine and Antarctic Studies University of Tasmania Hobart TAS Australia; ^6^ Département Adaptations du Vivant BOREA MNHN Paris France; ^7^ Department of Agriculture, Forestry and Fisheries South African Government Cape Town South Africa

**Keywords:** connectivity, individual‐based model, *Jasus paulensis*, *Jasus tristani*, lobster, population genetics

## Abstract

While there is now strong evidence that many factors can shape dispersal, the mechanisms influencing connectivity patterns are species‐specific and remain largely unknown for many species with a high dispersal potential. The rock lobsters *Jasus tristani* and *Jasus paulensis* have a long pelagic larval duration (up to 20 months) and inhabit seamounts and islands in the southern Atlantic and Indian Oceans, respectively. We used a multidisciplinary approach to assess the genetic relationships between *J. tristani* and *J. paulensis*, investigate historic and contemporary gene flow, and inform fisheries management. Using 17,256 neutral single nucleotide polymorphisms we found low but significant genetic differentiation. We show that patterns of connectivity changed over time in accordance with climatic fluctuations. Historic migration estimates showed stronger connectivity from the Indian to the Atlantic Ocean (influenced by the Agulhas Leakage). In contrast, the individual‐based model coupled with contemporary migration estimates inferred from genetic data showed stronger inter‐ocean connectivity in the opposite direction from the Atlantic to the Indian Ocean driven by the Subtropical Front. We suggest that the *J. tristani* and *J. paulensis* historical distribution might have extended further north (when water temperatures were lower) resulting in larval dispersal between the ocean basis being more influenced by the Agulhas Leakage than the Subtropical Front. As water temperatures in the region increase in accordance with anthropogenic climate change, a southern shift in the distribution range of *J. tristani* and *J. paulensis* could further reduce larval transport from the Indian to the Atlantic Ocean, adding complexity to fisheries management.

## Graphical abstract

Using a multidisciplinary approach combining genetic data (SNPs) with individual‐based modelling, we show that patterns of connectivity between rock lobsters *Jasus tristani* and *Jasus paulensis* changed over time in accordance with climatic fluctuations. Historical connectivity between the ocean basis was more influenced by the Agulhas Leakage (from the Indian to the Atlantic Ocean) while contemporary connectivity is stronger in the opposite direction from the Atlantic to the Indian Ocean, driven by the Subtropical Front.

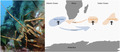

## 1 INTRODUCTION

One of the main objectives of fisheries management is to ensure sustainable exploitation and avoid stock depletion. Stocks generally respond predictably to fishery pressure and are able to recover under moderate levels of exploitation (Pitcher & Hart, 1982). However, an inadequate delineation of biological units and a lack of information on the source‐sink dynamics could compromise optimal exploitation, replenishment, and sustainability of stocks (Bernatchez et al., 2017). Despite this, fisheries management units are frequently based on territorial jurisdictions rather than on biological information (Reiss et al., 2009). In many cases, as with the model species used in our study, *Jasus tristani* and *Jasus paulensis*, management strategies are developed without basic connectivity and genetic data for the exploited species.

**FIGURE 1:**
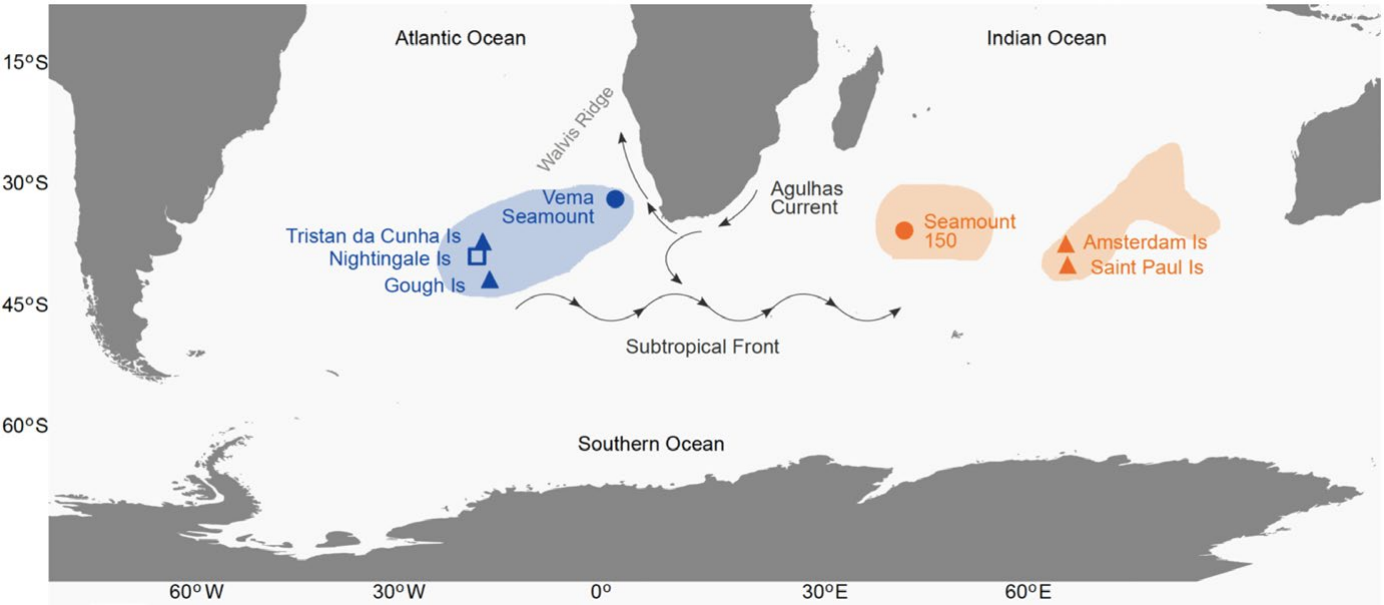
Sample locations and the approximate distribution range of *J. tristani* (blue) and *J. paulensis* (orange) (adapted from Booth, 2006). Triangles represent locations with both genetic data and individual‐based modeling data (the latter corresponding to release sites in the individual‐based model), circles represent locations with modeling data only, and the square represents a location with genetic data only

The Tristan rock lobster (*J. tristani*) and the St Paul rock lobster (*J. paulensis*) are distributed in a narrow latitudinal band (~25° to 47° S) in the Southern Atlantic and Indian Oceans, respectively (Booth, 2006; Figure 1). They are regularly found in shallow waters to a depth of 200 m (Holthuis, 1991) but occur as deep as 600 m, as demonstrated by recent (2019) catches during deep‐sea pots trials at Amsterdam Island (G. Duhamel, personal communication). Commercial harvesting of *Jasus tristani* occurs around the Tristan da Cunha Archipelago (Tristan da Cunha, Inaccessible and Nightingale Islands) and Gough Island (Roscoe, 1979; Scott, 2017). The resource is currently managed using Operational Management Procedures, and total allowable catches (TACs) are set annually for each island. Size limits and gear restrictions are also applied (Jeffs et al., 2013). While annual catches increased gradually from 331 t in 2001 to 442 t in 2010 (Jeffs et al., 2013), CPUE values have declined since 2005 (Glass, 2014). The intensive harvesting of the *J. tristani* population on Vema seamount in the 1960s led to the resource being considered “fished out” within two years (Heydorn, 1969) with little sign of recovery since then (von der Heyden et al., 2007). The *J. paulensis* fishery at St Paul and Amsterdam Islands (Duhamel, 1980; Pruvost et al., 2015; Vranckx, 1973) is operated from Réunion Island (landing place) using one fishing vessel with associated motorized small boats. The fishery (operational since 1949/50) was overexploited in the 1970s (Pruvost et al., 2015), which led to the introduction of a number of management measures including the setting of a total allowable commercial catch (TACC), a minimum legal size limit, a closed season and the protection of egg bearing females (Jeffs et al., 2013). The fishery has now stabilized at a TACC of about 400 tonnes with an increase in CPUE noted between 2001 and 2010 (G. Duhamel, personal communication). *J. tristani* and *J. paulensis* are currently treated as separate species for the purposes of fishery management with some acknowledgment of spatial differences in age and size structure. For example, the minimum landing size varies between the Tristan da Cunha islands and is set at 70 mm carapace length at Tristan and Nightingale, which gives females a chance to spawn before being captured (Scott, 2017). However, spatial genetic structure and source‐sink dynamics are not explicitly taken into account in the management. Failure to incorporate genetic information into management plans can result in local reduction of populations and in reduced overall productivity or decreasing CPUE values (Ovenden et al., 2015).

Advances in both genetic technologies and numerical modeling, and their integration into multidisciplinary approaches have improved our understanding of the complex factors that influence dispersal of marine species (Werner et al., 2007). Technological improvements and decreasing costs of sequencing (e.g., RAD‐Seq), as well as powerful bioinformatics tools and statistical software accessibility, have resulted in an increasing use of high‐resolution genomic markers (Davey et al., 2011). Markers, including single nucleotide polymorphisms (SNPs), have been applied in a range of marine animals, including nonmodel organisms, such as lobsters, and ﻿have proved to be useful for species delineation (e.g., Georges et al., 2018; Iguchi et al., 2019) and for estimating population connectivity (Silva, Villacorta‐Rath, et al., 2019). Similarly, individual‐based models (IBMs) have been used increasingly to infer larval dispersal and connectivity (Cowen et al., 2007; Gallego et al., 2007). Such models are generally forced by simulated fields from oceanographic models. Increases in computational capabilities have enabled the development of more sophisticated, higher‐spatial resolution oceanographic models, which in turn have allowed more detailed simulations of larval transport (Werner et al., 2007). Connectivity matrices from numerical models, with probabilities of larval dispersal between populations, have been combined with genetic distance and differentiation matrices in a seascape genetics approach (Selkoe et al., 2016). This multidisciplinary approach has enhanced our understanding of the complex drivers shaping population genetic structure (e.g., Benestan et al., 2016; Lal et al., 2017; Sandoval‐Castillo et al., 2018; Singh et al., 2018; Young et al., 2015). Combining genetics with oceanographic modeling ﻿is particularly valuable for the conservation and management of marine populations as it ﻿can identify ecological and biophysical characteristics associated with particular biological units or stocks, and any asymmetries in source‐sink dynamics (Selkoe et al., 2016 and references therein).

Rock lobsters have a long pelagic larval duration (PLD; potentially up to 20 months, which has been recorded for the closely related species *J. edwardsii*; Bradford et al., 2015), and therefore, it has been traditionally assumed that there would be high levels of gene flow between populations. However, the concept of “open” populations is no longer considered valid as barriers to dispersal have been identified in a wide range of marine animals with medium to long pelagic larval durations (e.g., Mattingsdal et al., 2020; Silva, Villacorta‐Rath, et al., 2019; Takeuchi et al., 2019; Thomas & Bell, 2013; Woodings et al., 2018). That said, species with a long PLD usually have low levels of genetic differentiation among populations as a result of large dispersal potential, large population sizes and high fecundity (Cowen & Sponaugle, 2009). Still, genetic data can also overestimate average dispersal distance as a few individuals may sporadically disperse long distances, thus reducing genetic differences among populations (Shanks, 2009). While the duration of an organism's larval phase and the extent of any environmental barriers to dispersal can be important predictors of dispersal potential, other life history characteristics, such as larval behavior and postsettlement survival, can also play a role in promoting or limiting dispersal (Cowen & Sponaugle, 2009; Moksnes et al., 2014; Villacorta‐Rath et al., 2018).

*Jasus tristani* and *Jasus paulensis* distribution ranges are located over 7,000 km apart but mitochondrial DNA (mtDNA) data have shown little to no genetic differentiation between these two species (Groeneveld et al., 2012; Ovenden et al., 1997). However, the authors highlight limitations and recommend increasing sample size and the amount of mitochondrial sequence data obtained from each individual as well as including nuclear sequences. In addition, George and Kensler (1970) noted that *J. tristani* and *J. paulensis* possess a significant morphological difference in the abdominal sculpture index. Although there is mixed evidence regarding the level of the divergence between *J. tristani* and *J. paulensis*, it is clear that this species pair is genetically distinct to all other *Jasus* species, including *J. lalandii*, which is distributed on the South African coast and situated only 3,000 and 4,000 km from the *J. tristani* and *J. paulensis* populations, respectively.

The recruitment dynamics and mechanisms of connectivity between *J. tristani* and *J. paulensis* remain largely unknown as is the linkage between these two geographically distant populations. As Groeneveld et al. (2012) emphasised, the percentage of larvae dispersing from the South Atlantic to the Indian Ocean has not yet been quantified. Processes such as ocean currents (Chiswell & Booth, 1999) and larval behavior in response to environmental orientation cues (Hinojosa et al., 2016) can contribute to complex source‐sink dynamics. Given the difficulty in accessing and studying these species in their natural habitat due to the isolation of the islands and challenging sea conditions, genetic and modeling approaches are valuable tools to help understand population dynamics and connectivity and inform fisheries management (Hinrichsen et al., 2011; Waples et al., 2008). Therefore, in this study we combined genetic data obtained from high‐resolution genomic markers (SNPs) with individual‐based modeling that incorporates flow fields from a large‐scale oceanographic model. We assessed the connectivity among *J. tristani* and *J. paulensis* populations and investigated the potential for contemporary and historical gene flow. We hypothesized that ocean currents directly influence the connectivity and genetic structure of *J. tristani* and *J. paulensis* populations. Because stocks are currently managed without any information on source‐sink dynamics, results from this study have application to ongoing fisheries management decision making.

## 2 METHODS

### 2.1 Genomic analyses

Tissue samples were collected in 2015 from three populations of *J. tristani* (Gough Island, Tristan da Cunha Island and Nightingale Island) and two populations of *J. paulensis* (Saint Paul Island and Amsterdam Island; Table 1). Tissue was stored in 70% ethanol before processing. Total genomic DNA was extracted using NucleoMag^®^ Tissue (Macherey‐Nagel) following the manufacturer's instructions. Library preparation and sequencing were conducted at Diversity Arrays Technology, Canberra, Australia and followed standard protocols of DArTseq™ genotyping technology (Kilian et al., 2012). Briefly, approximately 100 ng (2 µl) of each sample was digested with the restriction enzymes PtsI and SphI, and unique barcode sequences simultaneously ligated onto the ends of each resulting fragment as per Kilian et al. (2012). The PstI‐compatible adapter included an Illumina flow‐cell attachment sequence, a primer sequence and unique barcode, with the reverse SphI‐compatible adaptor contained in the flow‐cell attachment region. A minimum of 15% random technical replicates were included for downstream quality control. Each sample with fragments containing both PstI and SphI cut sites was amplified in independent PCR reactions using the following conditions: 94°C for 1 min then 30 cycles of 94°C for 20 s, 58°C for 30 s, 72°C for 45 s, and 72°C for 7 min. Samples were then purified using a Qiagen PCR clean‐up kit (Qiagen). Samples were checked visually on an agarose gel to ensure complete digestion and uniform range of fragment sizes. Using approximately 10 µl of each sample, samples were sequenced on a single flow‐cell lane on the Illumina HiSeq2500 for 77 cycles.

**TABLE 1:** Sample sizes for *Jasus tristani* and *J. paulensis* and summary statistics of genetic diversity per population using neutral 17,256 SNPs

Species	Population ID	Location	Coordinates (lat, lon)	Sample size	*H*_O_	*H*_E_	*A*_R_
*J. tristani*				31	0.183	0.505	1.83
	JTR_Tr	Tristan da Cunha Is	−37.05353, −12.2573	12	0.190	0.506	1.41
	JTR_NI	Nightingale Is	−37.42991, −12.48231	11	0.178	0.490	1.36
	JTR_Go	Gough Is	−40.3574, −9.93356	8	0.177	0.496	1.39
*J. paulensis*				67	0.176	0.272	1.87
	JPA_Am	Amsterdam Is	−37.7715, 77.5177	50	0.173	0.270	1.42
	JPA_SP	Saint Paul Is	−38.721, 77.56049	17	0.183	0.265	1.42
Total				98			

Abbreviations: *A*_R_, allelic richness; *H*_E_, expected heterozygosity; *H*_O_, observed heterozygosity.

Libraries were demultiplexed, and reads were filtered for overall quality (–c, –q and –r options) using *process_radtags* in STACKS v.2.0b9 (Catchen et al., 2013). The Stacks pipeline *denovo_map.pl* was executed to run each of the Stacks modules individually (*ustacks, cstacks, sstacks,* and *populations*).

To optimize the de novo assembly, we tested a range of parameters, including *m* (minimum stack depth) from 3 to 7 and *M* (distance allowed between stacks) and *n* (distance allowed between catalog loci) from 1 to 9, as recommended by Rochette and Catchen (2017) and Paris et al. (2017). The parameter test showed that *M* = 3 and *n* = 3 (the M and n parameters were kept equal) provided a balance between obtaining true polymorphism and introducing sequencing error (i.e., the number of widely shared loci plateaued at about *M* = 3 and *n* = 3) and that *M* = 3 and *n* = 3 was sufficient to stabilize the proportions of loci with 1–5 SNPs. Therefore, we retained *M* = 3 and *n* = 3 for the main analysis. The high coverage with the value of *m* = 3 (67×) and consistent results with *m* = 3 to *m* = 7 imply a true biological signal. As *m* = 3 also performs well for a broad range of data sets (Paris et al., 2017; Rochette & Catchen, 2017), we retained *m* = 3 for the main analysis. Reads were aligned de novo with each other and a catalogue of putative RAD tags was created (*cstacks* module). Putative loci were searched against the catalog (*sstacks* module) and further filtering was then conducted in the *populations* module.

Retained reads were present in at least 50% of samples within each species, detected in at least one species, had a rare allele frequency of at least 20%, and had no more than 2 alleles detected. Potential homologs were excluded by removing markers with heterozygosity >0.50 within samples following Hohenlohe et al. (2011). Individuals with more than 50% missing data were removed, resulting in a total of 98 samples (Table 1).

Allelic richness was estimated using *hierfstat* (version 0.04‐22) in R (Goudet, 2005). Pairwise F_ST_ values and respective p‐values were estimated using the software GenoDive version 3.0 (Meirmans & Van Tienderen, 2004). The R package *adegenet* version 2.1.1 was used to estimate observed and expected heterozygosity and for estimating membership probabilities using the compoplot() function in *adegenet* (Jombart, 2008). Genetic structure was further investigated with principal component analyses (PCA) using the R package *stats* version 3.5.1 (R Core Team, 2018). Outlier analyses were used to identify signatures of selection and two complementary approaches were conducted. In BayeScan (version 2.1), prior odds were set to 100 to minimize the chance of false positives with 5,000 pilot runs, followed by 100,000 iterations (5,000 samples, a thinning interval of 10, and a burn‐in of 50,000) (Foll & Gaggiotti, 2008). PCAdapt (version 4.0.3) is a hierarchical Bayesian model to determine population structure with latent factors and identifies outlier loci that contribute disproportionately to explaining each of the latent factors (Luu et al., 2017).

### 2.2 Migration estimates

To infer contemporary migration (first generation migrants), GeneClass2 (Piry et al., 2004) was used. In order for the GeneClass2 analysis to be computationally tractable, three subsets of 100 randomly chosen SNPs were tested (SNP subsets 1, 2 and 3). Migrants were detected by calculating the ratio of the likelihood computed from the population where the individual was sampled over the highest likelihood value among all population samples, including the population where the individual was sampled (Paetkau et al., 2004). The assignment threshold was set to 0.01, using a Bayesian method (Rannala & Mountain, 1997). Monte‐Carlo resampling method takes into account the sampling variance inherent to the limited size of the reference datasets to better control type I error rates (Paetkau et al., 2004). Monte‐Carlo resampling was based on the simulation of 10,000 individuals with a Type 1 error of 0.01 over all scored loci and individuals were assigned to the location with the highest likelihood.

Historical migration (from the current generation back to the most recent common ancestor) was estimated using a coalescent method implemented in MIGRATE 3.7 (Beerli & Felsenstein, 2001). In order for the MIGRATE analysis to be computationally tractable, three subsets of 100 randomly chosen SNPs were tested (SNP subsets 1, 2 and 3). Five independent runs were built for each subset of 100 SNPs and checked for convergence and the best run was selected. Runs were started under the SNP model using values of *Θ* (theta) and *M* (migration rate) calculated from *F*_ST_ values. The first 10,000 runs were discarded as burn‐in for each chain and runs consisted of sampling increments of 100 iterations with 10 replicates. Model convergence of MIGRATE analyses was confirmed using TRACER 1.7 (Rambaut et al., 2018). The number of migrants per generation was estimated using:

(1) xNm=Θ×M

where Θ (theta) is a constant value per population generated from *F*_ST_ calculations and *M* are mutation‐scaled immigration rates.

### 2.3 Individual‐based modeling

An individual‐based modeling (IBM) approach was used to investigate the transport and population connectivity of *Jasus tristani* and *J. paulensis*. The ocean flows used to drive the IBM were extracted from a global oceanographic model, specifically a 1/12° application of the Nucleus for European Modelling of the Ocean (NEMO) modeling framework, provided by the National Oceanography Centre, Southampton, UK. NEMO is a versatile modeling system that has been used for a range of modeling studies from regional to global scales, and the ability of the 1/12° configuration to represent key oceanographic features has been demonstrated (e.g., Deshayes et al., 2013; Marzocchi et al., 2015; Sérazin et al., 2015). Full details of the model may be found in Marzocchi et al. (2015). In summary, the model run was started from rest in 1978, initialized from the World Ocean Atlas (WOA) 2005 climatological fields. The simulation was forced by 6‐hourly winds, daily heat fluxes, and monthly precipitation fields. The model output fields used for this study comprised 5‐day mean velocities for 2000 to 2011, extracted for the region 70°W to 90°E, and 55°S to 0°S.

The mean flows from the oceanographic model were used to simulate the transport of Lagrangian particles representing the planktonic larvae of *Jasus* lobsters, using an established individual‐based model: the Hydrodynamics‐based Algorithm for Lagrangian simulations (HAL; Fox et al., 2006; Young et al., 2012). This model has been applied previously to studies of zooplankton transport (Young et al., 2014), and for the simulation of fish egg and larval transport (Fox et al., 2006; Young et al., 2015, 2018). Although HAL includes modules allowing the simulation of temperature‐dependent growth and mortality, diel vertical migration and directed swimming, there is insufficient information on the early life stages of *J. tristani* for inclusion of such complexity, and the model implementation was therefore comparatively simplistic. Specific model parameterizations for the simulation of *Jasus* sp. larvae considered spawning location and timing, depth distribution of larvae, and the duration of the pelagic larval stage. In summary, particles were advected at each model time step (30 min) according to the imposed three‐dimensional velocity field, using a second‐order Runge‐Kutta method, with additional horizontal and vertical diffusions included using a random‐walk approach (Dyke, 2001). Particles were released at six locations (JTR_Go, JTR_Tr, Ve, S1, JPA_Am, JPA_SP) with known populations of *Jasus tristani* and *J. paulensis* (Groeneveld et al., 2012; Figure 1), at model grid cells with a depth shallower than 1,000 m in order to capture the distribution of suitable spawning regions (to an observed depth of 600m). 100 particles per grid cell per day were released for the duration of the spawning period, August to October, for each of 10 years (2000–2009). Subsequent particle transport was simulated for a total possible planktonic period of 22 months, with particles randomly distributed within the upper 200m of the ocean. Successful metamorphosis to the puerulus stage and subsequent recruitment was allowed from 15 months, dependent on the arrival of a phyllosoma larva in a “recruitment zone” defined as model grid cells within 100km of one of the defined population sites. Due to limited observations of *J. tristani* and *J. paulensis*, the choice of parameterization was based on observations and prior modeling studies of *J. edwardsii* and *J. lalandii*, which have been studied in considerably more detail (e.g., Bradford et al., 2015; Bruce et al., 2007; Pollock, 1986; Stanley et al., 2015). However, the assumed spawning and planktonic periods agree with the limited observations of *J. tristani* phyllosoma and pueuruli (Vladimir Laptikhovsky, CEFAS, personal communication). Diel vertical migration (DVM) was not included in the representation of larval transport; there are no data on DVM in *J. tristani* or *J. paulensis* phyllosoma, and observations of DVM in *J. edwardsii* suggest that well‐defined DVM is only observed in late‐stage larvae (Bradford et al., 2005). In total, >5.7 million simulated particles were released over the 10 year study period.

For each simulation year, the percentage of larvae from each release site successfully recruiting to one of the defined recruitment zones was calculated, and the results were combined into connectivity matrices, ***M_t_***, for each year describing the proportion of individuals arriving in a destination population (rows) from a given source population (columns). Summary matrices were then calculated describing the mean and standard deviation in connectivity, and the number of years with nonzero connectivity. Particles transported among *J. tristani* and *J. paulensis* sites (and vice versa) were identified and their trajectories analyzed to identify dominant inter‐species connectivity pathways.

### 2.4 Demographic modeling

Several hypotheses of divergence modes were tested, aiming to identify the best divergence mode of *J. tristani* and *J. paulensis* and the direction of contemporary and historical migration. Six models were built considering possible scenarios: (SI) Strict Isolation, where the environment (e.g., sea level change and ocean currents) promoted allopatry; (IM) Isolation‐with‐Migration, with continuous gene flow since divergence; (AM) Ancient Migration, with an ancient gene flow event but recent isolation; (SC) Secondary Contact, with a recent gene flow event after past isolation; (PAM) Ancient Migration with two ancient gene flow events but recent isolation; and (PSC) Secondary Contact with two recent gene flow events after past isolation. All models were implemented allowing for asymmetric migration rates (m12, m21). In the models with two events of secondary contact (PAM and PSC), four parameters for migration rates were included (mA = m12 and mB = m21 in the first migration event and mC = m12 and mD = m21 in the second migration event) to determine changes in the direction of gene flow over time.

Demographic inference was performed using a folded joint site frequency spectrum (JSFS) in the diffusion approximation method implemented in the software ∂a∂i (Gutenkunst et al., 2009). The function *vcf2dadi in* the R package radiator (Gosselin, 2017) was used to create ∂a∂i SNP input files. Models were fitted using 20 replicate runs per model and the best model was retained. The Akaike information criterion (AIC) was used to perform comparisons among models (Sakamoto et al., 1986).

To compare among nested models of increasing complexity and address over‐parameterization issues, we used the comparative framework of Tine et al. (2014) by penalizing models which contain more parameters. For each species pair, a score was estimated for each model using:

(2) $$\mathrm{Score}=\frac{\left(\Delta_{\max}‐\Delta \mathrm{AIC}i\right)}{\Delta_{\max}}$$

where ∆_max_ corresponds to the difference in AIC between the worst and the best performing model (∆_max_ = AIC_max_ − AIC_min_) and ∆AIC_i_ = AIC_i_ − AIC_min_. Therefore, the worst model has a score of 0 and the best model has a score of 1. To evaluate the relative probabilities of the different models within each species pair, Akaike weights (*W*_AIC_) were also calculated following:

(3) $$W_{\mathrm{AIC}}=\frac{e^{\frac{‐(\Delta \mathrm{AIC}i)}{2}}}{\sum_{i=1}^{R}e^{\frac{‐(\Delta \mathrm{AIC}i)}{2}}}$$

where *R* corresponds to the total number of models considered (*R* = 6).

Demographic parameters were converted into indicative biological units, given the missing crucial information about mutation rate per generation in *Jasus* spp. The ancestral effective population size (*N*_ref_) before split for each species pair was estimated following:

(4) $$N_{\mathrm{ref}}=\frac{\theta }{4L\mu }$$

with *θ* being the optimal multiplicative scaling factor, μ the mutation rate (fixed at 2.3 × 10^−9^ mutations per site per generation; Flynn et al., 2017), and L the effective length of the genome explored:

(5) $$L=\frac{zy73}{x}$$

where *x* is the number of SNPs originally detected from y RAD tags of 73 bp present in the initial data set, and z the number of SNPs retained, following Rougeux et al. (2017). Estimated units in 2*N*_ref_ were converted to years assuming a generation time of 10 years (Pecl et al., 2009). Estimated migration rates were divided by 2*N*_ref_ to obtain the proportion of migrants in every generation.

## 3 RESULTS

### 3.1 Genomic analyses

A total of 575,777,486 quality‐trimmed sequencing reads were obtained, providing an average depth of coverage per individual over all SNPs of 58.8x. The catalog contained a total of 1,362,887 loci and after applying the different filtering steps, 18,879 SNPs were retained for further analyses. Although sample sizes at some locations were low (e.g., JTR_Go *n* = 8), the high number of SNP markers provide the necessary statistical power to accurately determine population genetic structure (Willing et al., 2012). Mean missing data across all individuals were 28% (from 16% to 49% per individual). Population JPA_Am showed the lowest values of observed heterozygosity, while JTR_Tr showed the highest values of observed heterozygosity. Overall *J. tristani* showed higher values of expected heterozygosity than *J. paulensis* (Table 1).

BayeScan identified 7 outlier SNPs and PCAdapt identified 1,623 outliers, including the 7 outliers identified by BayeScan. Due to the fact that we are investigating neutral processes (e.g., drift and migration) only the 17,256 neutral SNPs were used for the following analysis.

In general, pairwise F_ST_ values were low (0 < *F*_ST_ <0.05) with the highest values observed for the comparisons JTR_NI with *J. paulensis* populations (*F*_ST_ = 0.05), while the lowest pairwise *F*_ST_ values were observed for the comparisons of *J. paulensis* populations (*F*_ST_ = 0). Significant pairwise *F*_ST_ values were observed for all *J. tristani* and *J. paulensis* comparisons (*p* < .01; Table 2).

**TABLE 2:** Pairwise *F*_ST_ values (below diagonal) and *p*‐values (above diagonal) using 17,256 neutral SNPs

	JPA_SP	JPA_Am	JTR_Tr	JTR_Go	JTR_NI
JPA_SP	–	.276	.001	.001	.001
JPA_Am	.000	–	.001	.001	.001
JTR_Tr	**.024**	**.021**	–	.068	.010
JTR_Go	**.031**	**.028**	.011	–	.020
JTR_NI	**.034**	**.028**	.019	.033	–

Note

*F*_ST_ values where *p* < .01 are highlighted in bold.

**FIGURE 2:**
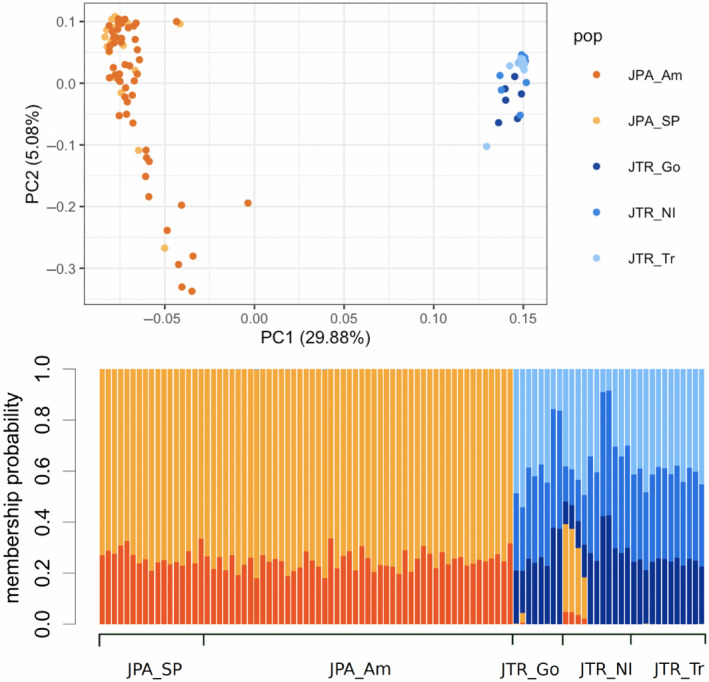
Principal component analysis (top) and membership probability plot (bottom) of *J. tristani* (JTR) and *J. paulensis* (JPA) using 17,256 neutral SNPs. JPA_SP: Saint Paul Island; JPA_Am: Amsterdam Island; JTR_Go: Gough Island; JTR_NI: Nightingale Island; JTR_Tr: Tristan da Cunha Island

The first principal component explained 29.88% of the total genetic variation and the principal component analysis grouped *J. tristani* and *J. paulensis* separately. Membership probability tests also indicated a clear differentiation between the genetic clusters with some evidence of admixture predominantly from *J. paulensis* to *J. tristani* (Figure 2).

### 3.2 Migration estimates

Contemporary migration estimates showed stronger connectivity from west to east, with 71 to 86% of first generation of migrants being transported from the Atlantic (*J. tristani*) to the Indian Ocean (*J. paulensis*). The total number of first generation migrants from *J. tristani* to *J. paulensis* varied from 12 to 17 while migrants from *J. paulensis* to *J. tristani* varied from 2 to 7 across the different SNP subsets (note that 61% of migrants were commonly identified across the three SNP subsets; Table 3). Historical migration estimates showed stronger connectivity from east to west, with 82%–86% of migrants per generation being transported from the Indian Ocean (*J. paulensis*) to the Atlantic (*J. tristani*). The total number of migrants per generation from *J. paulensis* to *J. tristani* varied from 62 to 107, while migrants from *J. tristani* to *J. paulensis* varied from 13 to 19 across the different SNPs samples (Table 3).

**TABLE 3:** Migration estimates using three subsets of 100 randomly chosen SNPs (SNP subsets sample 1, 2 and 3). (a–c) Contemporary migration estimated using GeneClass2 (first generation of migrants). (d–f) Historical migration inferred from Migrate‐*n* (number of migrants per generation)

		Source				
		JPA_Am	JPA_SP	JTR_Go	JTR_NI	JTR_Tr
Contemporary migration						
(a)						
Sink	JPA_Am	–	11	9	0	2
	JPA_SP	11	–	1	0	0
	JTR_Go	0	0	–	0	5
	JTR_NI	0	0	3	–	3
	JTR_Tr	0	2	3	2	–
(b)						
Sink	JPA_Am	–	19	2	4	1
	JPA_SP	11	–	1	1	3
	JTR_Go	2	0	–	4	2
	JTR_NI	1	0	0	–	4
	JTR_Tr	0	1	1	5	–
(c)						
Sink	JPA_Am	–	12	6	3	5
	JPA_SP	8	–	0	1	2
	JTR_Go	0	0	–	2	2
	JTR_NI	0	2	2	–	3
	JTR_Tr	4	1	1	2	–
Historical migration						
(d)						
Sink	JPA_Am	–	2.37	1.27	1.77	1.72
	JPA_SP	14.88	–	2.24	1.49	4.57
	JTR_Go	25.31	10.17	–	7.91	4.42
	JTR_NI	27.82	3.35	1.95	–	6.52
	JTR_Tr	10.98	2.80	1.94	3.05	–
(e)						
Sink	JPA_Am	–	2.95	1.81	2.50	2.28
	JPA_SP	22.24	–	4.34	2.77	5.46
	JTR_Go	17.63	3.15	–	11.99	5.78
	JTR_NI	39.42	5.63	9.15	–	6.40
	JTR_Tr	30.74	10.67	7.24	4.17	–
(f)						
Sink	JPA_Am	–	2.03	1.62	1.68	1.72
	JPA_SP	13.02	–	1.80	3.91	2.53
	JTR_Go	18.14	3.90	–	3.97	13.80
	JTR_NI	18.09	6.03	6.65	–	2.10
	JTR_Tr	12.93	3.20	1.71	10.36	–

### 3.3 Individual‐based modeling

**FIGURE 3:**
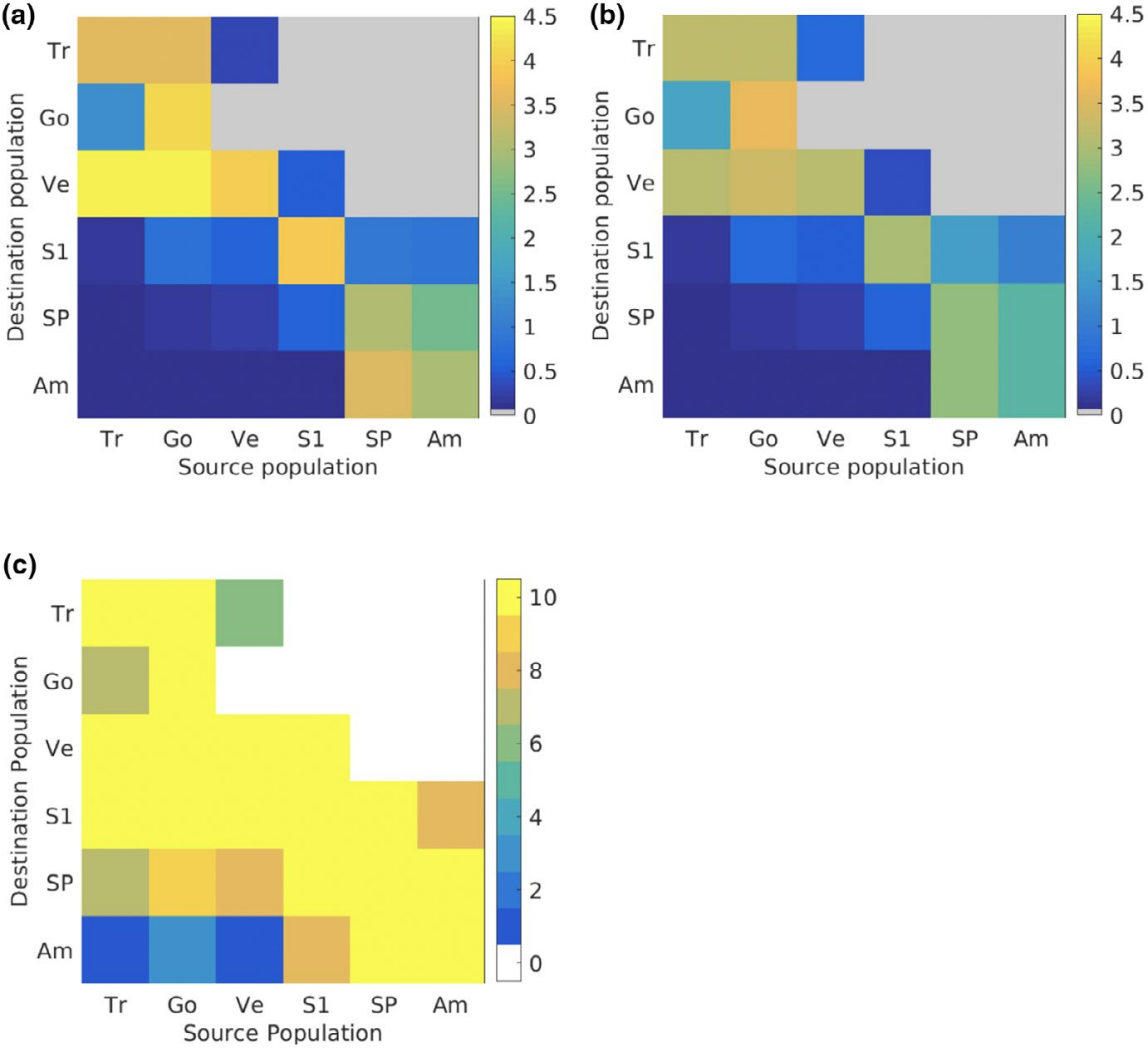
Simulated connectivity among island populations of *Jasus tristani* and *Jasus paulensis* expressed as: (a) mean connectivity as a percentage of particles from source populations (columns) successfully reaching destination populations (rows); (b) standard deviation in the annual connectivity; and (c) number of years with nonzero connectivity. Population identifiers are Tristan da Cunha archipelago (Tr), Gough Island (Go), Vema Seamount (Ve), Seamount 150 (S1), St Paul Island (SP) and Amsterdam Island (Am); see Figure 1 for locations. Mean and standard deviation in connectivity are plotted as log transformed percentages [log(10*x* + 1)] to better visualize the full range of values

The mean connectivity matrix (Figure 3a) suggests asymmetric dispersal of *Jasus* larvae both between and within the ocean basins. Predicted transport among islands in the Atlantic is strongly southwest to northeast, in accordance with the dominant anticyclonic flows of the South Atlantic gyre (Peterson & Stramma, 1991), with strong potential connectivity from both the Tristan da Cunha archipelago and Gough Island to Vema Seamount. Similarly, predicted transport from Gough Island to the Tristan da Cunha archipelago is much stronger and more consistent than in the opposite direction (>12 times larger on average, and connectivity in 10 years versus 7 years; Figure 3). Although there is a suggested dispersal of larvae from Vema Seamount to the Tristan da Cunha archipelago, it is weak and infrequent (nonzero connectivity in 6 years), and likely associated with the sporadic generation of eddies from the interaction of Agulhas rings with bathymetry (Schouten et al., 2000). Simulated dispersal of larvae among islands in the Indian Ocean is dominated by the anticyclonic circulation that predominates the large‐scale circulation in this region of the Indian Ocean and encompasses the Agulhas current that flows southwestwards along the coast of Africa (Stramma & Lutjeharms, 1997). Thus, there is bidirectional connectivity predicted among the *J. paulensis* populations at St Paul and Amsterdam Islands in all years (Figure 3c), and among these islands and Seamount 150 (belonging to the southwest Indian Ridge) in most years. However, there is stronger dispersal predicted from St Paul to Amsterdam than vice versa (>2.5 times larger), with the simulated percentage of particles from St Paul recruiting to Amsterdam Island greater than the percentage retained locally at Amsterdam Island (>1.5 times larger).

**FIGURE 4:**
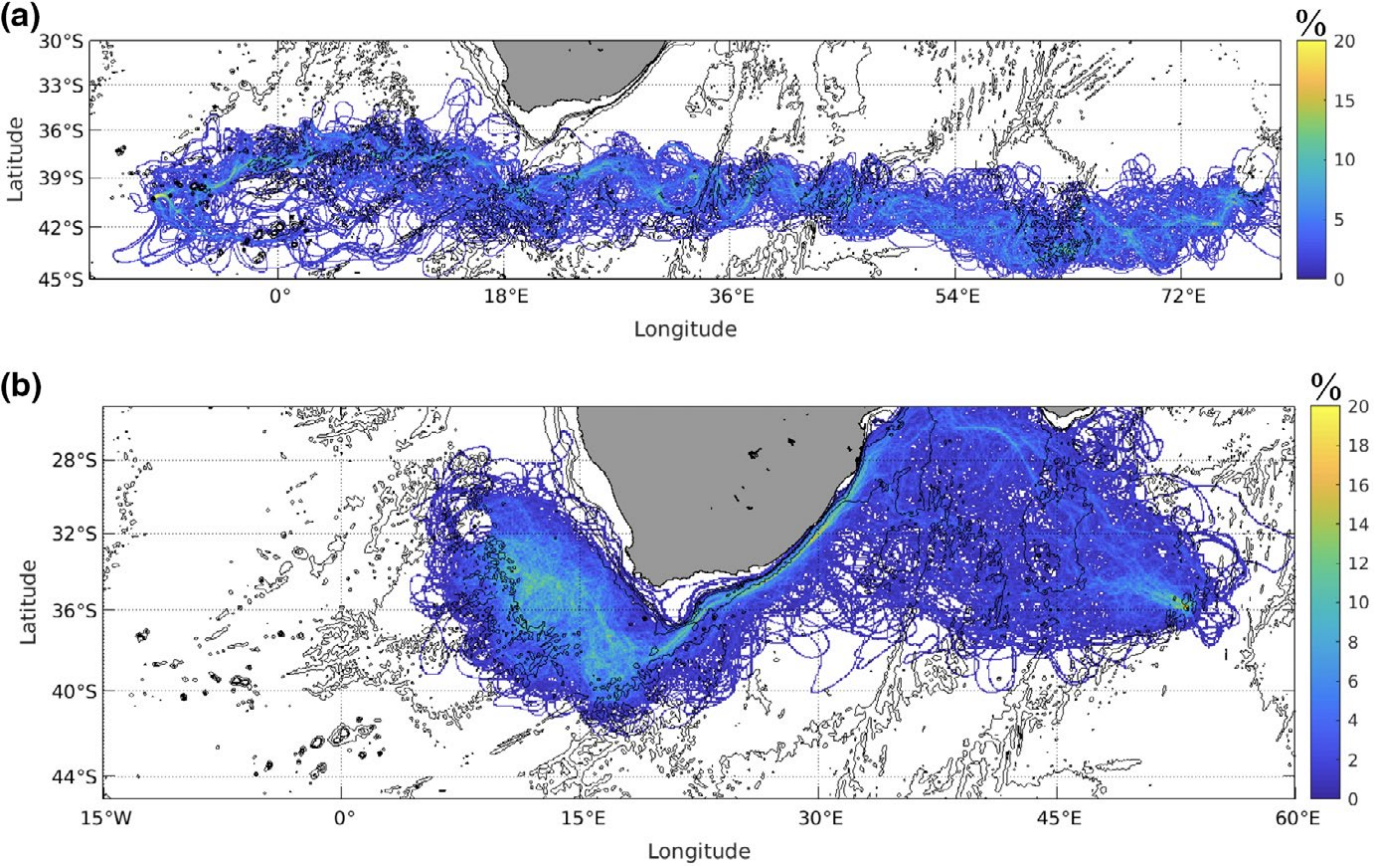
Dominant pathways of particles transported: (a) from Gough Island to St Paul Island (west to east); and (b) from Seamount 150 to Vema Seamount (east to west). Shading is the percentage of particles that reach the target region that pass through each model grid cell during the 10‐year simulation period, 2000–2009

The mean connectivity matrix shows finite values below the diagonal and predominantly zero values above the diagonal, indicative of potential inter‐ocean transport predominantly from west to east, from islands in the Atlantic toward those in the Indian. All islands in the Atlantic show some level of potential connectivity with islands in the Indian Ocean, with strongest and most consistent transport from Gough Island and Vema Seamount to Seamount 150 and St Paul Island. Transport from each of the three Atlantic islands to Seamount 150 is simulated in all 10 study years, with transport to St Paul Island from Gough Island and Vema Seamount in 9 and 8 of the 10 years, respectively. However, inter‐ocean transport is achieved by only a minority of the total number of particles released at the Atlantic Islands. Such particles escape the dominant anticyclonic circulation of the south Atlantic gyre in the southeast where the dynamic fronts are weak (Graham & De Boer, 2013), and are subsequently transported eastward in the Subtropical Front (Figure 4a). The connectivity matrix also suggests weak but consistent transport (all 10 years) from Seamount 150 to Vema Seamount. A small percentage of particles that are captured by the Agulhas Current in the southwest Indian Ocean continue into the Atlantic Ocean through Agulhas leakage and shedding of Agulhas rings (Lutjeharms, 2007). Turbulent mixing in the Cape Basin region (Boebel et al., 2003) then entrains particles in the south Atlantic gyre (Figure 4b). Thus, there is a potential weak east to west stepping stone connectivity from islands in the Indian Ocean to Vema Seamount. However, the model results show stronger inter‐ocean connectivity from west to east, thus suggesting that contemporary connectivity is predominantly through transport of Atlantic *J. tristani* larvae to Indian Ocean populations of *J. paulensis*.

### 3.4 Demographic modeling

**FIGURE 5:**
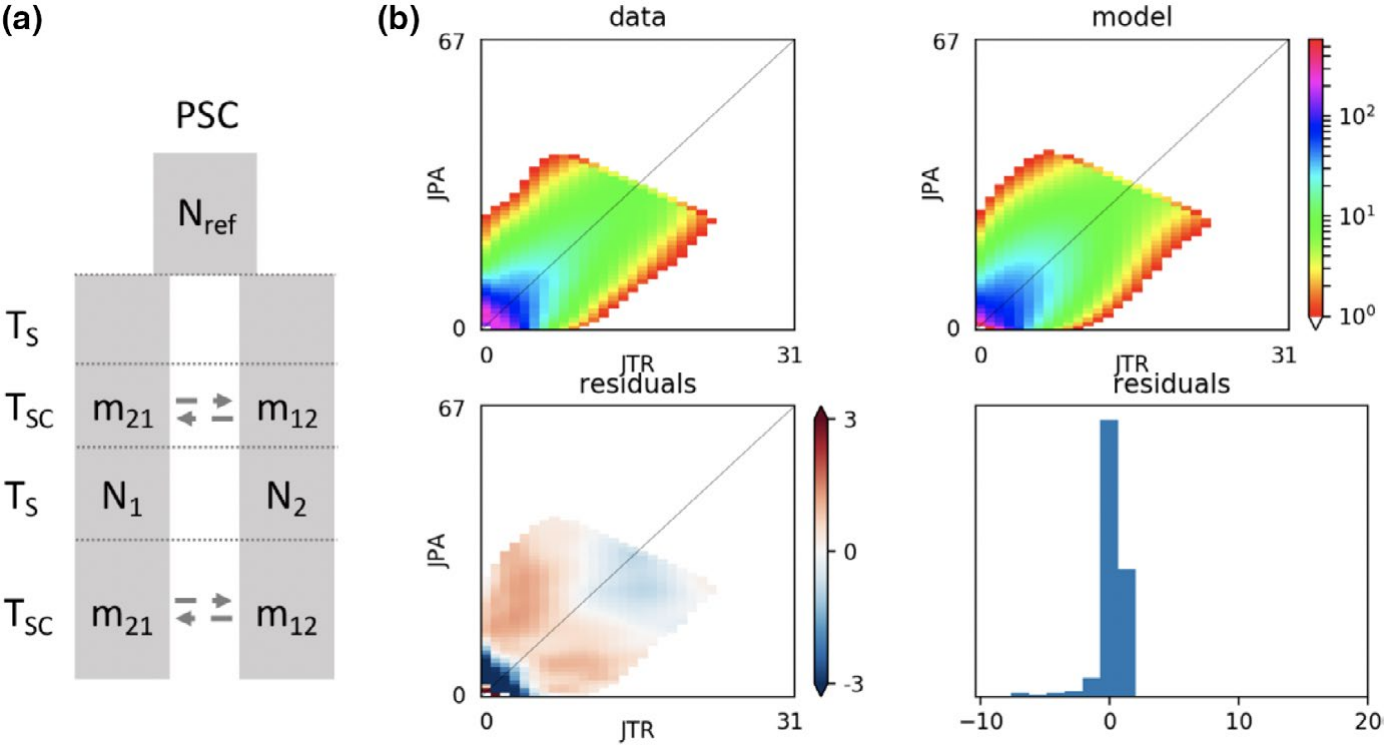
(a) Schematic of the best demographic model tested in the software ∂a∂i (Gutenkunst et al., 2009). PSC: Secondary Contact with two recent gene flow events after past isolation. *m*_21_ and *m*_12_: asymmetric migration rates; *N*_ref_: ancestral population size; *N*_1_, *N*_2_: population sizes after divergence; *T*_s_: the time of divergence in strict isolation; *T*_SC_: total time spent in secondary contact. (b) Joint site frequency spectrum of the best model (PSC) for *J. tristani* and *J. paulensis*

The demographic model with a scenario of two gene flow events after past isolation (PSC) provided better fits to the data with good predictions of the joint site frequency spectrum asymmetry (Log*L* = −2215.8, AIC = 4,447.6; Table 4, Figure 5b). The parameters of the demographic model for migration rates were in agreement with migration estimates based on genetic data (contemporary and historical) and with the individual‐based model (contemporary connectivity) showing stronger contemporary connectivity from west to east (transport of Atlantic *J. tristani* larvae to the Indian Ocean) and stronger historical migration from east to west (transport of *J. paulensis* larvae from the Indian to the Atlantic Ocean). Contemporary migration rates (m_JTR_‐>m_JPA_ = 18.3; m_JPA_‐>m_JTR_ = 3.34) were higher than historical migration rates (m_JTR_‐>m_JPA_ = 0.35; m_JPA_‐>m_JTR_ = 0.98; Table 4). Estimated time since divergence was 38,519 years [18,982–58,056]. Surprisingly, since divergence *J. tristani* and *J. paulensis* spent more time in strict isolation (*T*_S_ = 18,798 years [13,414–24,182]) than in secondary contact (*T*_SC_ = 461 years [0–8,443]).

**TABLE 4:** Results of model fitting for the different demographic scenarios: Strict Isolation (SI), Isolation‐with‐Migration (IM), Ancient Migration (AM), Secondary Contact (SC), Ancient Migration with two periods of ancient gene flow (PAM) and Secondary Contact with two periods of contact (PSC)

Model	*K*	Log*L*	AIC	Score	*W*_AIC_	Theta	*N*_1_	*N*_2_	m _JTR‐>JPA_	m _JPA‐>JTR_	m _JTR‐>JPA_	m _JPA‐>JTR_	*T*_S_	*T* _SC/AM_
PSC	8	−2,216	4,448	1.00	1.0	564	0.34	1.08	0.35	0.98	18.3	3.3	8.9	0.2
SI	3	−2,387	4,780	0.93	0.0	2,543	0.01	0.01	–	–	–	–	4^–04^	–
SC	6	−2,892	5,796	0.70	0.0	823	0.48	2.15	13.45	0.66	–	–	11.7	1.0
IM	5	−3,039	6,089	0.64	0.0	1,384	0.42	1.18	15.21	3.68	–	–	–	10.8
PAM	6	−4,497	9,007	0.00	0.0	901	0.83	1.61	15.13	18.82	–	–	3.8	0.1
AM	6	−4,266	8,544	0.10	0.0	312	2.36	2.64	11.29	12.89	–	–	11.6	0.2

Note

*K*, the number of free parameters in the model; LogL, best maximum likelihood estimates over 20 independent runs; AIC, Akaike Information Criterion; *W*_AIC_, Akaike weight of model i compared to the best model; Theta, theta parameter for the ancestral population before split (*θ* = 4*N*_ref_ *µ*), with *N*_ref_ being the effective size of the ancestral population, and *μ* the per‐site mutation rate per generation. *N*_1_, the effective size of population 1 before expansion; N_2_, the effective size of population 2 before expansion; *m*_12_, the neutral movement of genes from population 2 to population 1 in units of 2*N*_ref_ generations; *m*_21_, the neutral movement of genes from population 1 to population 2 in units of 2*N*_ref_ generations; *T*_S_, the time of divergence in strict isolation in units of 2*N*_ref_ generations; *T*_SC/AM_, the time of secondary contact/ancient migration in units of 2*N*_ref_ generations.

## 4 DISCUSSION

**FIGURE 6:**
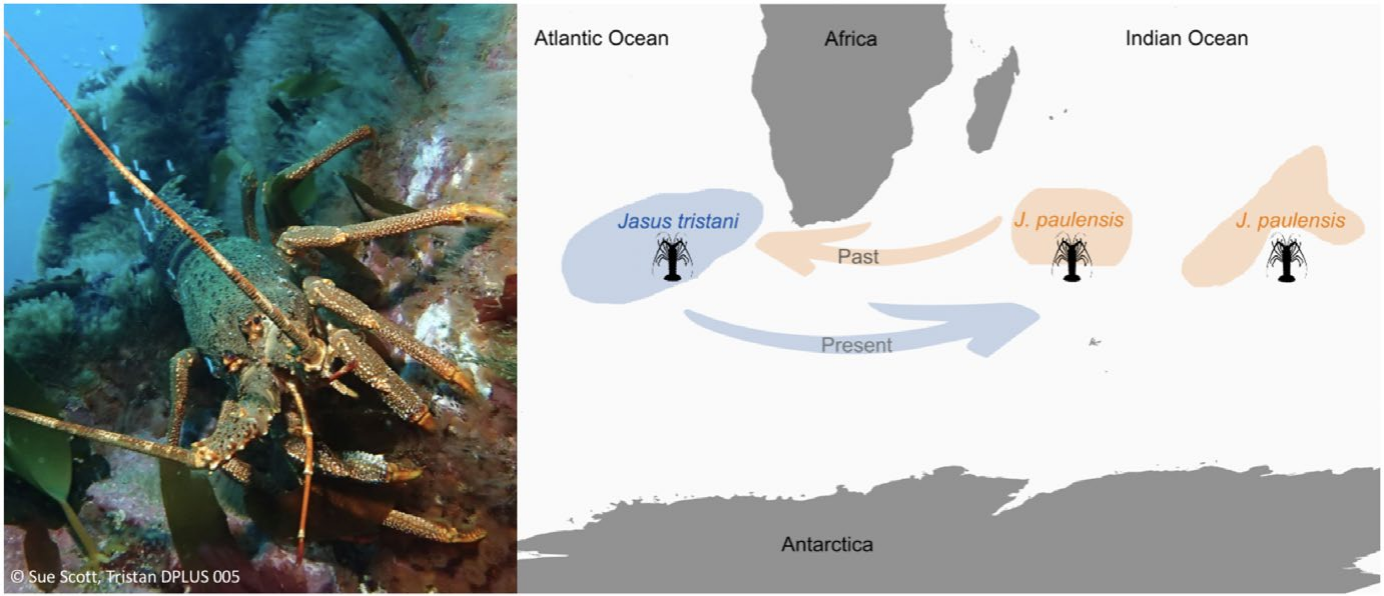
Tristan rock lobster, *Jasus tristani* (left) and illustration of changes over time in source‐sink dynamics in *J. tristani* and *Jasus paulensis* (right)

In this study, we found low but significant genetic differentiation between the rock lobsters *J. tristani* and *J. paulensis* using high‐resolution genomic markers. The individual‐based model and contemporary migration estimates inferred from genetic data showed stronger inter‐ocean connectivity from the Atlantic to the Indian Ocean (west to east), while historical migration estimates showed stronger connectivity from east to west (Figure 6). Results from this study have important implications for fisheries management, in particular by providing novel evidence of source‐sink dynamics across ocean basins and by giving insight into the possible effects of future environmental change on population connectivity.

### 4.1 Species delineation

In our study, *J. tristani* and *J. paulensis* individuals were clustered into separate groups, with low genetic differentiation and some evidence of admixture and gene flow. Pairwise values of differentiation between *J. tristani* and *J. paulensis* were low but significant values were observed (ranging from 0.021 to 0.034). These are consistent with F_ST_ values observed among populations of other lobster species. For example, American lobster (*Homarus americanus*) populations had a highest pairwise *F*_ST_ of 0.004 (Benestan et al., 2015), for the Eastern Rock Lobster (*Sagmariasus verreauxi*) the highest *F*_ST_ = 0.001 (Woodings et al., 2018), for the Southern rock lobster (*Jasus edwardsii*) the highest *F*_ST_ = 0.027 (Villacorta‐Rath et al., 2018), and for the European lobster (*Homarus gammarus*) the highest *F*_ST_ = 0.018 (Jenkins et al., 2019). Therefore, our study suggests high levels of genetic similarity between *J. tristani* and *J. paulensis*, which are higher than F_ST_ values observed among most populations in other lobster species.

Previous studies have shown mixed evidence regarding the taxonomic status of *J. tristani* and *J. paulensis*. George and Kensler (1970) noted that *J. tristani* and *J. paulensis* possess a significant morphological difference in the abdominal sculpture index. On the other hand, using mtDNA (cytochrome oxidase I gene), Groeneveld et al. (2012) showed little to no genetic differentiation between *J. tristani* and *J. paulensis* and suggested they should be synonymized. Using genome‐wide data (2,596 SNPs) Silva et al. (2021) showed evidence of admixture between *J. tristani* and *J. paulensis* but, in accordance with this study, results showed that these species spent more time in strict isolation (*T*_S_ = 10,524 years [±2,346]) than in secondary contact (*T*_SC_ = 1,941 years [±618]) since initial divergence. Also, the Tristan da Cunha and Gough Islands and the Amsterdam and St. Paul Islands have been grouped in the same zoogeographic province, called the West Wind Drift Islands Province as there is evidence of the same fish species that are endemic to both regions (Collette & Parin, 1991). Therefore, the similarities in habitat type in this zoogeographic province combined with high potential for dispersal may have facilitated gene flow between *J. tristani* and *J. paulensis*. However, using a high number of genomic markers (17,256 SNPs), our study suggests low but significant differentiation among species.

### 4.2 Historical and contemporary connectivity

In this study, historical migration estimated from the genetic data show stronger connectivity from east to west (from the Indian to the Atlantic Ocean), which is reflected in the higher genetic diversity in the Indian Ocean, and in the admixture analyses (showing introgression of *J. paulensis* into *J. tristani*). Groeneveld et al. (2012) also detected higher levels of genetic diversity in the Indian Ocean populations suggesting that these (*J. paulensis*) are the putative ancestral populations. However, levels of genetic diversity can also reflect higher effective population sizes as there are probably more populations of *J. paulensis* distributed throughout the southern Indian Ocean, whereas *J. tristani* may be less widely distributed in the southern Atlantic (Booth, 2006).

Compared with other lobster species (e.g., *Panulirus* spp. and *Palinurus* spp.), *J. tristani* and *J. paulensis* are distributed throughout a narrow range of temperatures (approximately 8–18°C; Booth, 2006). Therefore, with lower temperatures in the last glacial maximum (LGM), the distribution range of *J. tristani* and *J. paulensis* might have shifted north while tracking their thermal niche. In addition, decreasing oceanic temperatures and lower sea levels would have created newly available shallow benthic habitat for lobsters, allowing the colonization of northern areas (e.g., along the Walvis Ridge; Schaaf, 1996). Due to the high degree of uncertainty in paleoceanographic studies and the range of different proxies used, there is still debate around the position of the Subtropical Front (STF) and the volume of Agulhas leakage during the LGM (Kohfeld et al., 2013). The most geographically comprehensive study (Gersonde et al., 2003) suggests that the STF may have shifted equatorward by ~3° in the Atlantic; however, such a shift relates to the movement of specific isotherms and not temperature gradients, and it is known that identifying fronts, and their associated currents, by means of a single isotherm can be misleading (Chapman et al., 2020). Multiple proxies have been used to infer a reduction in Agulhas leakage at the LGM (e.g., Bard & Rickaby, 2009; Peeters et al., 2004). However, Franzese et al. (2006) concluded that the general positions of currents had not changed, and seasonal characteristics of some micropaleontological proxies suggested a stronger Agulhas leakage during winter (Martínez‐Méndez et al., 2010). With this uncertainty in mind, we hypothesize that a more northerly distribution of *J. tristani* and *J. paulensis* at the LGM reduced the influence on larval dispersal of the eastward currents associated with the STF, while the westward Agulhas leakage had a relatively larger influence. Thus, there were likely higher levels of gene flow from *J. paulensis “*populations” to *J. tristani*. Finally, Groeneveld et al. (2012) showed that *J. paulensis* underwent a population expansion between 14,000 and 118,000 years ago while von der Heyden et al. (2007) estimated 12,000–99,000 years ago for *J. tristani* in the Atlantic. Therefore, the last glaciation event and interglacial period might have had substantial impacts on these populations.

Contemporary connectivity estimated from the genomic markers and the individual‐based model suggested flow from west to east (Atlantic to Indian Ocean). This was also reflected in the admixture analyses but to a lesser extent than historical migration estimates. This pattern could be the result of a shorter period of contemporary connectivity (influenced by the STF), than historical connectivity, perhaps due to more northward historical distribution patterns.

Contemporary connectivity inferred from the individual‐based model corresponds to annual connectivity matrices and describes the transport of planktonic larvae among sites for spawning in one year. However, although any particles failing to reach a suitable recruitment site are assumed to die, the model does not include a consideration of instantaneous mortality, which is not well known, and would be expected to significantly reduce the simulated connectivity. Previous estimates of apparent mortality for similar species have quoted values of 98% (*J. edwardsii*; Lesser (1978) and 99.61% (*J. lalandii*; Lazarus, 1967). The connectivity matrices also take no account of postsettlement survival, which is likely to be lower for new recruits than for the existing adult population. Such factors are important to consider when interpreting the connectivity matrices in the context of population genetics. In addition, transfer of genetic variability among sites is cumulative and the single event connectivity matrices described here do not resolve stepping stone transport (e.g., Young et al., 2015).

### 4.3 Current implications for fisheries management and considerations for the future

Our study identified a potential model of source‐sink dynamics among *J. tristani* and *J. paulensis* populations. Currently, larval transport occurs mainly from the Atlantic to the Indian Ocean (influenced by the STF) but the Agulhas Current and Leakage also facilitate a stepping stone connectivity in the opposite direction, from the Indian to the Atlantic Ocean. This complex connectivity scenario has important implications in recruitment for fisheries management of the benthic stocks and how it could be altered under future environmental conditions.

Under such a model, fishing‐induced depletion of some populations (source) may reduce the connectivity and recruitment in other (sink) populations. This may have demographic consequences for populations and compromise productivity or recovery (e.g., Lindegren et al., 2014; Silva, Macdonald, et al., 2019). For example, the particle‐tracking model and genetic results show that contemporary inter‐ocean connectivity occurs predominantly from the Atlantic (*J. tristani*) to the Indian Ocean (*J. paulensis*). Therefore, depletion of *J. tristani* populations can not only reduce local recruitment but, to a lesser degree, the recruitment of *J. paulensis* populations as well. In addition, reduced source populations could compromise the recovery of sink populations in cases where source populations are suddenly severely depleted due a catastrophic event or events. Information on spatial genetic structure and source‐sink dynamics should be used to design more biologically relevant management units at the population level and more integrated management strategies (Reiss et al., 2009). The level/rate of larval exchange indicated in this study suggests that, while exchange is important, recruitment in each of these group populations is likely to be derived from local resident females and the protection of breeding stock in each island group is therefore important. An integrated management strategy maximizing egg production would not only benefit the fisheries at each of the island groups and across ocean basins but would also ensure exchange among island groups and, importantly, be vital for recruitment to the various seamount populations. Future studies should incorporate genetic data from seamount populations, which are likely to have smaller population sizes and be more dependent on recruitment from other (source) populations. Finally, as connectivity can change over time and with fluctuating environmental conditions and egg/larval numbers, the temporal stability of the genetic structure of populations should also be assessed periodically.

Shifts in species distribution ranges have been frequently reported as a result of climate change (Poloczanska et al., 2013), in particular, for marine species with large dispersal potential. For example, there is some evidence that the eastern rock lobster *Sagmariasus verreauxi* is becoming more common further south along the Tasmanian east coast (Robinson et al., 2015). In accordance with predicted increasing temperatures, *J. tristani* and *J. paulensis* may shift their distribution range further south while tracking their thermal niche. In fact, there has been an occurrence of *J. paulensis* in the Kerguelen Islands (Holthuis, 1991) albeit highly questionable (G. Duhamel, personal communication). Within increasing temperatures the subantarctic Islands of the Indian Ocean such as Prince‐Edwards, Crozet or Kerguelen Islands can become suitable habitat for these species. This would have implications for both fisheries management and the benthic ecology in these areas. A decrease in recruitment in the current fishery areas is likely to result in reduced local TACs and landings and force fishery vessels to travel longer distances to exploit new stocks. In addition, the shift of lobsters to more southern areas could lead to marked changes in the benthic ecology and community structure in these areas (Blamey & Branch, 2012; Cockcroft et al., 2008).

Ocean currents can strongly influence the genetic structure of species with long pelagic larval duration. Shifts in circulation patterns due to environmental change may affect connectivity, adding complexity to fisheries management (e.g., Potts et al., 2014). Ocean circulation is projected to change worldwide by the end of the century with changes in the intensity and position of western boundary currents including the Agulhas Current, the retroflection of which is projected to intensify and shift to the southeast (van Gennip et al., 2017; Popova et al., 2016). There is, however, considerably uncertainty regarding projected changes to circulation patterns in the Agulhas region (e.g., Tim et al., 2019), in part due to the relatively coarse spatial resolution of the presently available climate simulations. Nonetheless, an intensification of the Agulhas retroflection, in combination with a southwards shift in distribution of *Jasus* species, could result in reduced east to west connectivity of *J. paulensis* and *J. tristani*.

Our study provides new insights into the recruitment dynamics and connectivity of marine species with high dispersal potential and highlights the importance of incorporating multidisciplinary approaches into management decisions. Population connectivity and source‐sink dynamics are currently not explicitly incorporated in the management practices of *J. paulensis* and *J. tristani* fisheries. Accounting for such source‐sink dynamics, for example by applying management measures to maintain particular levels of biomass in each population in relation to that population's likely role in supporting recruitment, may result in higher average recruitment and a more successful fishery.

## CONFLICT OF INTEREST

All authors declare that they have no competing interests.

## AUTHOR CONTRIBUTION

**Catarina N. S. Silva:** Conceptualization (equal); Data curation (equal); Formal analysis (equal); Methodology (equal); Visualization (equal); Writing‐original draft (lead); Writing‐review & editing (lead). **Emma F. Young:** Conceptualization (supporting); Data curation (equal); Formal analysis (equal); Funding acquisition (lead); Writing‐original draft (supporting); Writing‐review & editing (equal). **Nicholas P. Murphy:** Conceptualization (equal); Funding acquisition (equal); Writing‐original draft (supporting); Writing‐review & editing (supporting). **James J. Bell:** Conceptualization (equal); Funding acquisition (supporting); Writing‐original draft (supporting); Writing‐review & editing (supporting). **Bridget S. Green:** Conceptualization (equal); Funding acquisition (equal); Writing‐review & editing (supporting). **Simon A. Morley:** Funding acquisition (equal); Writing‐original draft (supporting); Writing‐review & editing (supporting). **Guy Duhamel:** Data curation (equal); Writing‐original draft (supporting); Writing‐review & editing (supporting). **Andrew C. Cockcroft:** Writing‐original draft (supporting); Writing‐review & editing (supporting). **Jan M. Strugnell:** Conceptualization (lead); Funding acquisition (lead); Supervision (lead); Writing‐original draft (equal); Writing‐review & editing (equal).

## Data Availability

Sequencing data, pipelines for de novo assembly, genetic structure, migration estimates and demographic inference analyses are available at: https://github.com/CatarinaNSSilva/lobsters_climate_driven_dynamics.
